# Protective Effects of *Baccharis dracunculifolia* Leaves Extract against Carbon Tetrachloride- and Acetaminophen-Induced Hepatotoxicity in Experimental Animals

**DOI:** 10.3390/molecules19079257

**Published:** 2014-07-02

**Authors:** Túlio P. Rezende, José Otávio do A. Corrêa, Beatriz J. V. Aarestrup, Fernando M. Aarestrup, Orlando V. de Sousa, Ademar A. da Silva Filho

**Affiliations:** 1 Núcleo de Identificação e Pesquisa em Princípios Ativos Naturais - NIPPAN, Faculdade de Farmácia, Universidade Federal de Juiz de Fora, Rua José Lourenço Kelmer, s/n – Campus Universitário, Bairro São Pedro, CEP36036-900, Juiz de Fora, MG, Brazil; 2 Laboratório de Bioatividade Celular e Molecular, Universidade Federal de Juiz de Fora, Rua José Lourenço Kelmer, s/n – Campus Universitário, Bairro São Pedro, CEP36036-900, Juiz de Fora, MG, Brazil; 3 Laboratório de Imunopatologia e Patologia Experimental, Universidade Federal de Juiz de Fora, Rua José Lourenço Kelmer, s/n – Campus Universitário, Bairro São Pedro, CEP36036-900, Juiz de Fora, MG, Brazil; 4 Laboratório de Farmacologia de Produtos Naturais, Universidade Federal de Juiz de Fora, Rua José Lourenço Kelmer, s/n – Campus Universitário, Bairro São Pedro, CEP36036-900, Juiz de Fora, MG, Brazil

**Keywords:** *Baccharis dracunculifolia*, Brazilian green propolis, acetaminophen, carbon tetrachloride, hepatoprotective

## Abstract

In this work we investigated the *in vivo* protective effects of *Baccharis dracunculifolia* leaves extract (BdE) against carbon tetrachloride (CCl_4_)- and acetaminophen (APAP)-induced hepatotoxicity. Total phenolic content, total flavonoid content, antioxidant DPPH radical scavenging activity, and HPLC analysis were performed. Our results showed that pretreatment with BdE significantly reduced the damage caused by CCl_4_ and APAP on the serum markers of hepatic injury, AST, ALT, and ALP. Results were confirmed by histopathological analysis. Phytochemical analysis, performed by HPLC, showed that BdE was rich in *p*-coumaric acid derivatives, caffeoylquinic acids and flavonoids. BdE also showed DPPH antioxidant activity (EC_50_ of 15.75 ± 0.43 μg/mL), and high total phenolic (142.90 ± 0.77 mg GAE/g) and flavonoid (51.47 ± 0.60 mg RE/g) contents. This study indicated that *B. dracunculifolia* leaves extract has relevant *in vivo* hepatoprotective properties.

## 1. Introduction

Reactive oxygen species (ROS) are generated spontaneously in cells during metabolism and are implicated in the aetiology of different degenerative diseases, such as heart diseases, stroke, rheumatoid arthritis, diabetes and cancer [1]. Oxidative stress is also the main cause of liver diseases, and plant extracts with antioxidant activity have received special attention as possible preventive and therapeutic agents, since they are able to decrease the production and/or to eliminate produced ROS [2,3]. Chemicals, such as carbon tetrachloride (CCl_4_) and acetaminophen (APAP) produce liver toxicity associated to oxidative stress, causing necrosis of hepatocytes and, as a result, releasing enzymes, such as aspartate aminotransferase (AST), alanine aminotransferase (ALT), and alkaline phosphatase (ALP) into the circulating blood [4].

Previous studies have shown that the use of some natural compounds found in teas, fruits, and vegetables is associated with low risk of several degenerative diseases [1]. In addition, natural antioxidant substances may act against both CCl_4_- and APAP-toxicity, decreasing production and/or eliminating the free radicals responsible for hepatic damage [5]. Consequently, there is a great deal of interest in plants, beverages and foods that contain antioxidants and health-promoting phytochemicals as potential therapeutic agents [1].

One such plant is *Baccharis dracunculifolia* D.C. (Asteraceae), popularly known as “alecrim do campo” and “vassoura”, which is used as a tea with anti-inflammatory properties in Brazilian folk medicine [6] and as a hepatoprotective [7]. The aerial parts of *B. dracunculifolia* have also been used in farms to clean mud ovens before making cookies, which thus acquire the aroma of this plant [6]. In this way, the essential oil of *B. dracunculifolia* has commercial value due to its strong lasting exotic aroma, and its potentially wide range of applications, such as an additive in cosmetics, foods and agrochemicals [8].

Besides its use in traditional medicine, *B. dracunculifolia* is the most important botanical source of Southeastern Brazilian propolis, which due to its colour is called as green propolis [9]. Propolis is a natural resinous substance collected by honeybees (*Apis mellifera*) from buds and exudates of plants to be used as a protective barrier in the beehive that displays many biological activities, such as antioxidant [10], antibacterial [11] and hepatoprotective activity against both CCl_4_ [12] and APAP-induced damage [13].

Currently, because of their biological activities, Brazilian green propolis and *B. dracunculifolia* leaves are used in foods and beverages, especially in Brazil and Japan, aiming to improve health and to prevent several diseases, such as inflammation and cancer [14]. Recently, it was verified that a glycolic extract of *B. dracunculifolia* leaves exhibited *in vitro* antioxidant activity [2], which supposes that it may have some health beneficial effects *in vivo*. However, the extent of its protective effects *in vivo* depends on the bioavailability of its active compounds for intestinal absorption, metabolism, and subsequent interaction with target tissues. In this line, in the present study, we investigated the activity of *B. dracunculifolia* leaves extract against CCl_4_- and APAP-induced liver damage, which have not been reported.

## 2. Results and Discussion

### 2.1. HPLC Analysis of Hydroalcoholic Extract of B. dracunculifolia (BdE)

**Figure 1:**
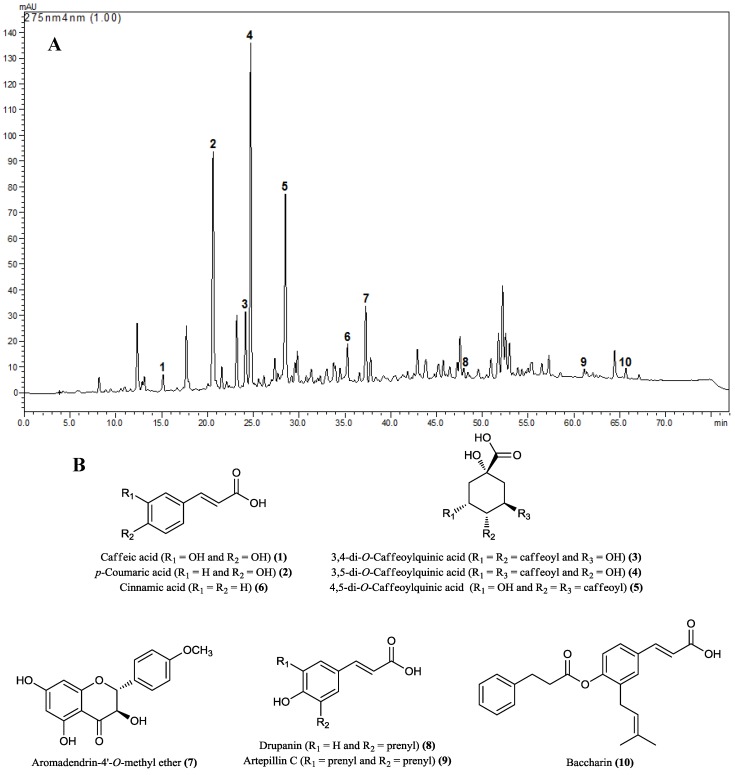
(**A**) Representative HPLC chemical profile of BdE recorded at 275 nm. (**B**) Chemical structures of major compounds identified from BdE: caffeic acid (**1**), *p*-coumaric acid (**2**), 3,4-di-*O*-caffeoylquinic acid (**3**), 3,5-di-*O*-caffeoylquinic acid (**4**), 4,5-di-*O*-caffeoylquinic acid (**5**), cinnamic acid (**6**), aromadendrin-4'-*O*-methyl ether (**7**), drupanin (**8**), artepillin C (**9**), and baccharin (**10**).

The HPLC analysis of BdE allowed the identification of caffeic acid (**1**), *p*-coumaric acid (**2**), 3,4-di-*O*-caffeoylquinic acid (**3**), 3,5-di-*O*-caffeoylquinic acid (**4**), 4,5-di-*O*-caffeoylquinic acid (**5**), cinnamic acid (**6**), aromadendrin-4'-*O*-methyl ether (**7**), drupanin (**8**), artepillin C (**9**), and baccharin (**10**) as the major identified compounds (Figure 1A, B).

### 2.2. DPPH Free Radical Scavenging Assay, Total Phenolic Content and Total Flavonoid Content

DPPH radical scavenging activities of BdE (which ranged from 5 to 50 μg/mL) increased in a concentration-dependent manner (18.13% to 93.28% of inhibition) with an EC_50_ of 15.75 ± 0.43 μg/mL, while rutin showed an EC_50_ of 8.71 ± 0.24 μg/mL. Total phenolic content of BdE was 142.90 ± 0.77 mg GAE/g of BdE. Total flavonoid content obtained from BdE was 51.47 ± 0.60 mg of rutin equivalent (RE)/g of BdE.

### 2.3. CCl_4_-Induced Hepatotoxicity

**Figure 2:**
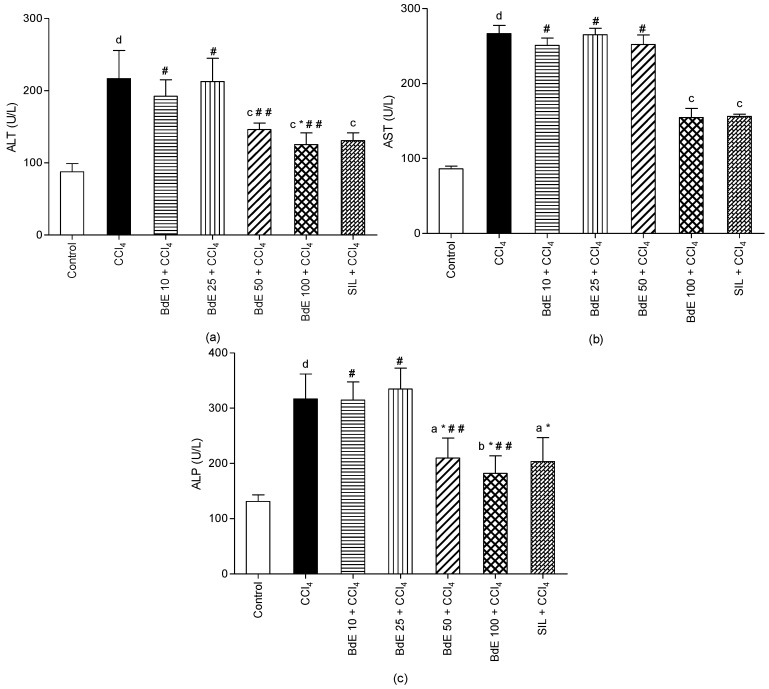
Effect of BdE on mice intoxicated with carbon tetrachloride (CCl_4_). Levels of liver enzymes AST (**a**), ALT (**b**) and ALP (**c**).

The effects of BdE at four dose levels (10, 25, 50 and 100 mg/kg bw, p.o.) on serum marker enzymes in CCl_4_-induced hepatic injury are shown in Figure 2.

Hepatic injury induced by CCl_4_ caused significant (*p* < 0.001) rise in marker enzymes ALT, AST, and ALP when compared with the normal control group. On the other hand, administration of BdE (100 mg/kg bw, p.o.) significantly (*p* < 0.001) attenuated the increased levels of the serum enzymes produced by CCl_4_, and caused a subsequent recovery towards normalization like that of silymarin (100 mg/kg bw, p.o.) treatment. The activity of BdE (100 mg/kg bw, p.o.) is comparable to the silymarin, a known hepatoprotective drug.

Data are expressed as the mean ± S.D. for six animals per group. ^a^ *p* < 0.05; ^b^ *p* < 0.01; ^c^ *p* < 0.001 (*vs.* CCl_4_ group); ^d^ *p* < 0.001 (*vs.* Control group); N.S. ^*^ *p* > 0.05 (*vs.* Control group). Notes: ^#, # #^ Non-significant differences between BdE groups.

### 2.4. APAP-Induced Hepatotoxicity

**Figure 3:**
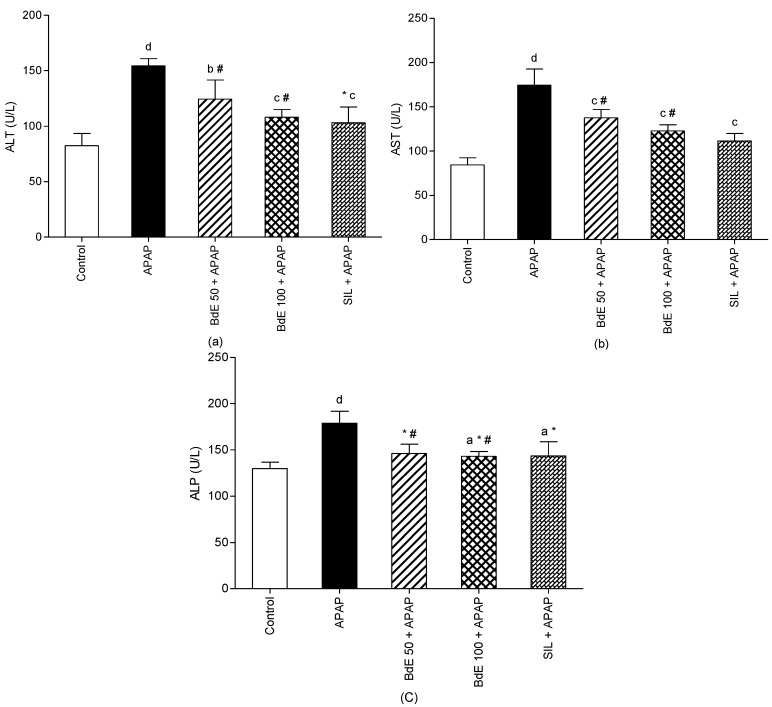
Effect of BdE on rats intoxicated with acetaminophen (APAP). Levels of liver enzymes AST (**a**), ALT (**b**), ALP (**c**).

Administration of APAP (600 mg/kg bw, p.o.) caused significant (*p* < 0.001) liver injury, as demonstrated by the increase of serum marker enzymes of ALT by 87.36%, AST by 106.88%, and ALP by 37.86% compared to control group (Figure 3).

Administration of BdE (50 and 100 mg/kg bw, p.o.) significantly attenuated the increased levels of the serum enzymes produced by APAP, like the silymarin (100 mg/kg bw, p.o.) treatment. Non-significant differences were observed between groups treated with 50 and 100 mg/kg of BdE.

Data are expressed as the mean ± S.D. for six animals per group. ^a^ *p* < 0.05; ^b^ *p* < 0.01; ^c^ *p* < 0.001 (*versus* CCl_4_ group); ^d^ *p* < 0.001 (*versus* Control group); N.S. ^*^ *p* > 0.05 (*versus* Control group). Notes: ^#^ Non-significant differences between BdE groups.

### 2.5. Histopathological Studies

**Figure 4:**
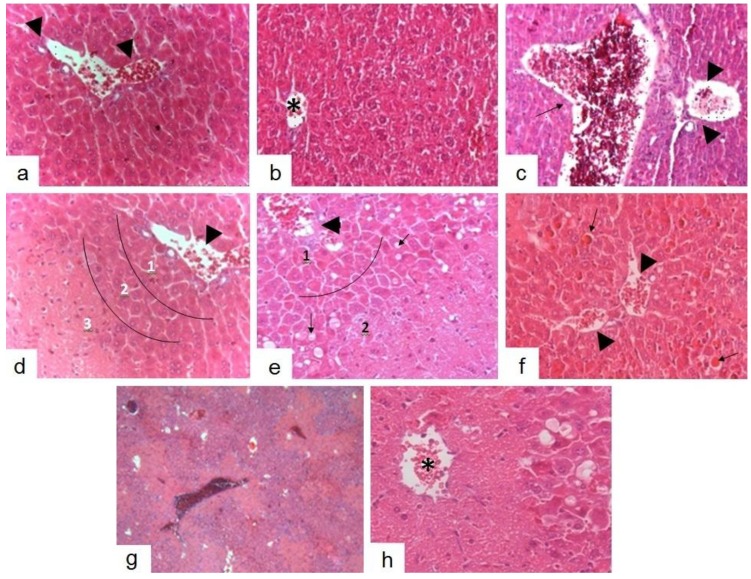
Protective effects of BdE pretreatment on CCl_4_-induced hepatotoxicity in rats. Photomicrographs of liver sections from animals treated with: (**a**) silymarin (100 mg/kg), (**b**) BdE (100 mg/kg), (**c**) vehicle, (**d**) BdE (10 mg/kg), (**e**) BdE (25 mg/kg), (**f**) BdE (50 mg/kg), (**g**) and (**h**) CCl_4_ (5.0 mL/kg) (hepatotoxicity control). Portal triad (arrowhead). Central venule (asterisk). Hepatic vein (arrow). Delimitations of zones 1, 2 and 3 (line). Staining H.E. Magnification 400×.

Histopathological analysis of liver sections from groups treated with silymarin (100 mg/kg bw) (Figure 4a), BdE (100 mg/kg bw) (Figure 4b), and normal control (vehicle) group (Figure 4c) exhibited characteristics consistent with normality, in which the capillary network can be seen between Remak’s fibres. Hepatocytes of zones 1, 2 and 3 (taking as reference the portal triad and central venule) displayed intensely eosinophilic cytoplasm, typical glycogen granules and intracytoplasmic basophilic round nuclei and were strongly basophilic. Occasionally, in the group treated with BdE (100 mg/kg bw), cells were observed in zone 3 and 2 with bulkier cores and dispersed chromatin as well as discreet hyaline accumulation. In all regions, including such areas, normality was noted in capillary pattern and maintenance of trabecular bone (Figure 4b). Liver sections from the group treated with BdE (10 mg/kg bw) showed intensely eosinophilic areas with loss of trabecular cytoarchitecture typical around the zone 3 of the hepatic lobes, corresponding to coagulation necrosis, occasionally expanding to the same region in adjacent lobes (Figure 4d). Additionally, in zone 2, surrounding the necrotic area, isolated hepatocytes under apoptosis and necrosis were observed and zone 1 showed characteristics compatible with normality (Figure 4d). The group treated with BdE (25 mg/kg bw) displayed confluent necrosis in zone 3 and part of zone 2 in the most lobes observed, with occasional continuing this process to centroacinares regions of neighboring lobes (Figure 4c). Also, some hepatocytes in zone 1 exhibited consistent changes with degenerative accumulations, without loss of radial architectural arrangement, capillary changes and acinar cell organization (Figure 4c). Samples from the group treated with BdE (50 mg/kg bw) displayed maintenance of trabecular radial cytoarchitecture on all lobe areas (Figure 4f). In zone 3, isolated hepatocytes with bulkier core and dispersed chromatin were observed. Furthermore, in zone 1, hyaline accumulation was slightly more significant of that observed in the group treated with BdE (100 mg/kg bw) (Figure 4b). Displayed glycogen storage was compatible with normality. Hepatotoxicity control (CCl_4_ group) exhibited vascular congestion associated with damaged areas of radial trabecular structure and lobular structure loss (Figure 4g). Hepatocytes with degeneration features, necroinflammatory “spotty necrosis” and several phases of apoptosis were observed in zone 2, associated with dilatation of sinusoidal capillaries and Disse’s space (Figure 4h). Beyond that, in none of the samples were areas of diffuse inflammatory infiltrate, polymorphonuclear or mononuclear, as well as fibrosis, observed. The BdE (100 mg/kg bw)-treated group showed normal liver architecture as it possessed higher hepatoprotective action (Figure 4b).

### 2.6. Discussion

*B. dracunculifolia* secondary metabolites are collected by bees (*Apis mellifera*) to produce Brazilian green propolis, which is of great importance for the food and pharmaceutical industries [10,11]. Besides, B. dracunculifolia is a medicinal plant used as an infusion to prevent diseases [6,7]. In the present study, the capability of *B. dracunculifolia* leaves extract (BdE) to protect against CCl_4_- and APAP-induced hepatotoxicity was investigated.

Our results demonstrated that BdE possess antioxidant activity and high contents of phenolics and flavonoids. The DPPH free radical test is a decolorization assay, which measures the relative antioxidant abilities of compounds or extracts to scavenge the free radical (DPPH) generated, which makes this assay system one of the most commonly used antioxidant test methods [15]. Phenolic compounds are ubiquitous in plants and are characterized by hydroxylated aromatic rings and known to act as antioxidants [16]. Our results demonstrated that BdE possesses significant amounts of flavonoids and mainly phenolic compounds, which may be responsible for the antioxidant activity of BdE. Previous phytochemical studies of *B. dracunculifolia* reported the identification of phenolic acid derivatives and flavonoids as the major compounds of its aerial parts [17,18]. Recently, it was reported that a glycolic extract of *B. dracunculifolia*, rich in phenolics, also possesses *in vitro* antioxidant activity [2].

Considering the chemical composition of BdE, individual phenolic compounds were determined by HPLC analysis, which demonstrated that phenolic acids (caffeic acid, *p*-coumaric acid, cinnamic acid, drupanin, baccharin and artepillin C), hydroxycinnamic acids (3,4-di-*O*-caffeoylquinic acid, 3,5-di-*O*-caffeoylquinic acid and 4,5-di-*O*-caffeoylquinic acid) and flavonoid compounds (aromadendrin-4'-methyl ether) were predominant in the composition of BdE, which is in agreement with previous investigations [19]. Among the major compounds identified in BdE, caffeic acid (**1**) and its derivatives **3**, **4** and **5** have shown potent antioxidant activity in the DPPH assay. It was also reported that *p*-coumaric acid (**2**) and its prenylated derivatives drupanin (**8**) and artepillin C (**9**) possess strong antioxidant activity [20]. These data allow us to suggest that the phenolic compounds present in BdE could be responsible for its antioxidant activity. Also, we hypothesize that the major metabolites of BdE might protect the liver from CCl_4_ and APAP-induced damages.

Then, in order to evaluate the hepatoprotective effect of BdE, a CCl_4_-induced hepatotoxicity model was employed first. This experimental model has been used extensively to investigate hepatoprotective activity on different experimental animals [21]. The hepatotoxicity induced by CCl_4_ is mainly due to its metabolite CCl_3_^−^, a free radical that alkylates cellular proteins and other macromolecules with a simultaneous attack on polyunsaturated fatty acids. In the presence of oxygen, lipid peroxides are produced, leading to liver damage [21], characterized by fatty liver, cirrhosis and necrosis [22]. Oxidative stress is considered to play a prominent causative role in many diseases, including liver damage [4]. Oxidative stress is the state of imbalance between the level of antioxidant defense system and production of ROS, such as superoxide radical (O^2−^), hydroxyl radical (OH^−^) and hydrogen peroxide (H_2_O_2_). Thus, the antioxidant activity or the inhibition of the generation of free radicals is important for protection against CCl_4_-induced hepatotoxicity [21,22]. In addition, in CCl_4_-induced hepatotoxicity, the extent of hepatic damage is assessed by the increased level of cytoplasmatic enzymes (ALT, AST and ALP), which leads to leakage of large quantities of enzymes into the blood circulation and could be regarded as an index of the liver parenchymal cells damage [4]. Clinically, the general strategy for prevention and treatment of the CCl_4_-induced hepatotoxicity includes reducing the production of reactive metabolites [23]. Also, CO_2_-induced hyperventilation has been used for the treatment of intoxication with CCl_4_, since that liver metabolizes normally less than 1% of the ingested CCl_4_, and the remainder is excreted by the lungs [24,25].

The treatment with BdE was capable of significantly and dose-related attenuation of the increased levels of the serum enzymes (ALT, AST and ALP), produced by CCl_4_-induced hepatotoxicity, like silymarin, a known hepatoprotective drug [5]. The protective effects exerted by BdE against CCl_4_-induced hepatotoxicity were further confirmed by conventional histopatological assessment. It was observed that CCl_4_-intoxicated mice, pretreated with BdE, showed normal liver architecture, demonstrating the hepatoprotective action of BdE.

In an attempt to verify the protective effects of BdE against APAP-induced liver damage, only the most active doses of BdE found in the CCl_4_-induced hepatotoxicity model (50 and 100 mg/kg bw) were employed. Acetaminophen has been used extensively during the last 40 years as a model toxicant, and one of the most popular models to test potentially hepatoprotective agents, especially natural products [26]. APAP toxicity is initiated by the metabolism of a small fraction of the dose by cytochrome P450 enzymes, in order to form *N*-acetyl-*p*-benzoquinone imine (NAPQI). An increasing amount of NAPQI reacts with protein sulfhydryl groups, causing covalent adduction of cellular proteins. In addition, NAPQI binds to mitochondrial proteins, leading to an initial mitochondrial oxidative stress, which is an important early event in the mitochondrial dysfunction after APAP overdose. Also, ROS have been proved to associate with the intoxication by APAP, and the major ROS of APAP toxicity in mice is superoxide (O_2_^−^) [27]. In addition, AST, ALT, and ALP activities are well-documented biochemical markers of hepatic dysfunction after APAP-induced toxicity. Clinically, the antioxidant *N*-acetyl-L-cysteine (NAC) is known to restore the glutathione depleted by NAPQI during APAP metabolism, and therefore, NAC is used in the human clinical treatment of APAP-induced toxicity [23].

Thus, the administration of BdE significantly reduced the activities of AST, ALT and ALP compared to the APAP-induced toxicity group. Then, it is suggested that, *in vivo*, BdE may scavenge free radicals and protect mitochondrial, endoplasmatic reticulum, and plasma membranes from damage induced by free radicals. Several studies have indicated that natural compounds exhibited strong antioxidant activity that could act against CCl_4_- and APAP-induced liver damage [5,28]. Almost all of those studies demonstrated the mechanism by which phenolic compounds prevent hepatotoxicity was due to their antioxidant properties [29]. Phenolic compounds may also contribute directly to antioxidant action, because of their redox properties, which allow them to act as reducing agents, hydrogen donors and singlet oxygen quenchers [29]. Among the identified compounds in BdE, dicaffeoylquinic acids **3**, **4** and **5** showed hepatoprotective effects in cultured hepatocytes against CCl_4_-toxicity. In addition, aromadendrin-4'-*O*-methyl ether (**7**) and baccharin (**10**) had been proved to possess hepatoprotective effect against D-GalN/TNF-α-induced cell death in primary cultured mouse hepatocytes [20,30].

Therefore, we found that treatment with BdE markedly inhibits CCl_4_- and APAP-induced liver damages as evidenced by decreased serum activities of AST, ALT and ALP, which was supported by histopathological examinations. Then, the protective effects of BdE may be due, at least in part, to the increased antioxidant activity and free radical scavenging effects of phenolic compounds and flavonoids present in BdE. Finally, considering that there are differences between experimental animal and human responses to CCl_4_- and APAP-induced liver damages, more studies are necessary in order to find out the clinical relevance of the protective effects of BdE.

## 3. Experimental

### 3.1. Chemicals

DPPH free radical (1,1-diphenyl-2-picrylhydrazyl), carbon tetrachloride (CCl_4_), acetaminophen (APAP), silymarin, caffeic, *p*-coumaric and cinnamic acids were purchased from Sigma-Aldrich (St. Louis, MO, USA). Artepillin C was purchased from Wako Pure Chemical Industries Co. (Osaka, Japan) and di-*O*-caffeoylquinic acid derivatives were obtained from PhytoLab (Vestenbergsgreuth, Germany). Aromadendrin-4'-*O*-methyl ether, drupanin and baccharin were previously isolated and identified as described [19] and kindly donated. Automation reagents for aspartate aminotransferase (AST), alanine aminotransferase (ALT), and alkaline phosphatase (ALP) were purchased from Wiener Lab Group (Rosario, Santa Fe, Argentina). Folin-Ciocalteu reagent, rutin and gallic acid were purchased from Merck (Darmstadt, Germany). All other chemicals were of analytical-reagent grade.

### 3.2. Plant Material

Leaf buds of *Baccharis dracunculifolia* De Candole were freshly collected at Federal University of Juiz de Fora – UFJF (Juiz de Fora, Minas Gerais State, Brazil) in February 2011. The plant material was previously authenticated by P.L. Viana (Federal University of Minas Gerais), and a voucher specimen (CESJ 47.482) was deposited at the Leopoldo Krieger Herbarium, Institute of Biological Sciences, Federal University of Juiz de Fora, Juiz de Fora, Brazil.

### 3.3. Preparation of the Hydroalcoholic Extract of B. dracunculifolia (BdE)

Leaves were dried and stabilized in a greenhouse of air circulating at 40 °C for 48 h and powdered to a fine grade in a blender. Air-dried, powdered leaves (210 g) were exhaustively extracted with ethanol/H_2_O (9:1 v/v), at room temperature by maceration. The filtered extract was concentrated under vacuum below 45 °C to furnish 72 g of the crude hydroalcoholic extract of *B. dracunculifolia* leaves (BdE), which was sealed in a glass bottle and stored at 2 °C until used.

### 3.4. HPLC Analysis of BdE

HPLC analysis of the BdE was undertaken by using Shimadzu Prominence Liquid Chromatography system (Shimadzu Corporation, Kyoto, Japan) equipped with a CBM-20A controller, four LC-20AT pumps, a SPD-M20A diode-array detector model and Shimadzu LC solution version 1.21 software controller. A Shimadzu Shim-Pack CLC-ODS column (4.6 × 250 mm, particle diameter of 5 μm, pore diameter of 100 Å) was used. The mobile phase consisted of a buffer solution in pump A (water–formic acid 0.1% v/v, pH 2.7) and methanol in pump B. The elution was undertaken using a linear gradient of 20%–95% of B in 77 min at a flow-rate of 0.8 mL/min and the detection performed at 275 nm. BdE was diluted with 5 mL of methanol (HPLC grade) in 10 mL volumetric flasks, sonicated for 40 min, and filled to volume with Milli-Q water. The sample was filtered through a 45 μm filter before analysis. All compounds were identified by comparison to their retention times and by co-elution with available authentic chromatographic standards [17], according to the previously reported method [2].

### 3.5. DPPH Radical Scavenging Activity

Free radical-scavenging activity of BdE was estimated using the stable DPPH radical (DPPH) assay [31]. BdE stock solution was prepared in ethanol at a concentration of 1 mg/mL and diluted to obtain different concentrations (5–30 μg/mL). One milliliter of ethanolic DPPH solution (100 μM) was added to 2.5 mL of ethanolic extract solution at various concentrations. Forty minutes later, the absorbance was read at 517 nm, at room temperature, in triplicate, with a Shimadzu UV-VIS 1800 spectrophotometer. The percentage of antioxidant activity (%AA) was calculated according to the equation:

%AA = 100 − [(sample absorbance/control absorbance) × 100]

Thus, the amount of antioxidant required to decrease by 50% the initial concentration of DPPH was calculated by interpolating the absorbance of the sample against a standard curve of rutin and expressed as 50% efficient concentration (EC_50_).

### 3.6. Total Phenolic Content

Total phenolic content was determined according to the Folin-Ciocalteu method [32] with some modifications. Two hundred milliliter of BdE solution (1 mg/mL) was added to 5.0 mL of Folin-Ciocalteu reagent and mixture was kept at room temperature for 8 min. Four milliliters of 7.5% aqueous sodium carbonate was added to the mixture. Water was added to adjust the final volume to 15 mL. After 30 min in the dark, at room temperature, the absorbance was read at 778 nm, in triplicate, with a Shimadzu UV-VIS 1800 spectrophotometer. Total phenolic content was determined by interpolating the absorbance of the sample against a standard curve of gallic acid (1.0–5.0 µg/mL) and expressed as mg of gallic acid equivalent per gram of BdE (mg GAE/g).

### 3.7. Total Flavonoid Content

Total flavonoid content was measured using the aluminum chloride colorimetric method as described by Dewanto, *et al.* [33] with minor modification. Briefly, 5.0 mL of BdE ethanolic solution (10 mg/mL) was added into a centrifuge tube with 2.0 mL of dichloromethane, 3.0 mL of distilled water and centrifuged for 3 min. A 2.0 mL aliquot of the upper layer previously prepared was mixed with 600 μL of glacial acetic acid, 10 mL of pyridine-ethanol solution (2:8 v/v) and 2.5 mL of 8% ethanolic aluminum chloride were added to a 25 mL volumetric flask. The total volume of the mixture was adjusted to 25 mL with distilled water. After 15 min at room temperature, the absorbance was read at 420 nm, in triplicate, with a Shimadzu UV-VIS 1800 spectrophotometer. Total flavonoid content was expressed as mg of rutin equivalent per gram of BdE (mg RE/g) from a calibration curve of rutin standard solution (2.0–30.0 μg/mL).

### 3.8. Animals

Male Swiss albino mice (weighing 20–25 g, 5–6 weeks) and male Wistar albino rats (weighting 200–250 g, 8–12 weeks) were used for the experiments. All animals were obtained from the Animal Center of Biology and Reproduction (CBR) of Federal University of Juiz de Fora and held in the experimentation room of Laboratory of Pharmacology of Natural Products, UFJF, Brazil. All animals were grouped and housed in polyacrylic cages (29 × 18 × 16 cm), with beds of sawdust, feed hoppers and water bottle. Moreover, animals were kept at 25 ± 2 °C, relative humidity 55% ± 5%, with light/dark cycles of 12 h (7:00 to 19:00), received balanced feed Nuvilab Rodents (Nuvital Nutrients, Colombo, Brazil) and water *ad libitum*. These conditions were maintained for an acclimatization period of 7 days and during the treatment protocol. At the end of the experimental period, animals were sacrificed by withdrawing blood from the inferior vena cava under light diethyl ether anesthesia. The serum was obtained by centrifugation and stored at −80 °C until analysis and the liver was also removed and stored at −80 °C until use. Animals were treated in accordance with the guiding principles established by the Brazilian College of Animal Experimentation and the rules of the Council for International Organizations of Medical Sciences. Experiments were authorized by the Ethical Committee for Animal Care of Federal University of Juiz de Fora (protocols 017/2011 and 034/2012).

### 3.9. CCl_4_-Induced Hepatotoxicity Model

Mice were randomly divided into seven groups of six animals each, according to a previously reported protocol [34]. In the test groups I-IV, animals were given 10, 25, 50 and 100 mg per kilogram-body weight (bw)/day, orally, using gavage. These doses were based on previous *in vivo* studies with extracts of *B. dracunculifolia* [6]. Group V (SIL) received silymarin (100 mg/kg bw/day) and groups VI (CCl_4_) and VII (normal control) only received vehicle (1% Tween 80/0.9% normal saline solution). All administrations were conducted for 7 days. On the seventh day, 2 h after the last oral administration, a single dose of carbon tetrachloride (intraperitoneally, 5.0 mL/kg bw, diluted to 2% in mineral oil) was applied intraperitoneally to groups I-VI. The vehicle control (group VII) received equivalent amount of mineral oil alone. Animals were sacrificed 16 h after the CCl_4_ dose for assessment of liver function and histopathological examinations.

### 3.10. APAP-Induced Hepatotoxicity Model

The experiment was conducted according to method previously described [35] with minor modifications. Rats were randomly divided into five groups of six animals each. Test groups I and II received BdE at doses of 50 and 100 mg/kg bw, orally, respectively. Silymarin (100 mg/kg bw) was administered to group III (SIL) and groups IV (APAP) and V (normal control) only received vehicle (1% Tween 80/0.9% normal saline solution). One hour after the treatment (groups I-V), a single dose of acetaminophen (orally, 600 mg/kg bw, diluted in vehicle) was given to groups I-IV. The vehicle control (group V) only received equivalent amount of saline. Animals were sacrificed 24 h after the APAP administration for assessment of liver function.

### 3.11. Assessment of Liver Function

Serum biochemical parameters aspartate aminotransferase (AST), alanine aminotransferase (ALT) and alkaline phosphatase (ALP) were measured in an Automation system 3000 plus BT (Wiener lab Group) using commercial kits acquired of Wiener lab. All assays were performed at the Clinical Analysis Laboratory of UFJF, which is certificated by the Brazilian Society of Pathology and Laboratory Medicine.

### 3.12. Liver Histopathological Examinations

The material from total hepatectomy was cleaved along, with long axis of the section from the major hepatic blood vessels and its branches in the right lobe, according to surgical procedure described by Nolan and Leibowitz [36]. After routine histological processing, the staining hematoxylin and eosin (HE) was performed [37]. Sections of 5 μm in thickness were analyzed on Zeiss microscope (Hallbergmoos, Germany) in increment of 400×, in all its extension to general descriptive histopathological evaluation. The double blind analysis was performed by two different observers with experience and training in histopathology. From this assessment were selected in 400× magnification, representative areas of each sample to capture digital computerized AxioVision^®^ (Zeiss, Berlin, Germany), using digital camera coupled to an optical microscope.

### 3.13. Statistical Analysis

Statistical analysis of the results were performed applying one-way analysis of variance (ANOVA) followed by Tukey post test using GraphPad Prism version 5.04 for Windows software (GraphPad Software, San Diego, CA, USA). All results are expressed as mean ± standard deviation (S.D.) and values of *p* < 0.05, *p* *<* 0.01, and *p* *<* 0.001 were considered statistically significant.

## 4. Conclusions

Our results demonstrated, for the first time, that the oral treatment with BdE is effective for prevention of both CCl_4_- and APAP-induced liver damage, similarly to Brazilian propolis [12,13]. Then, this study demonstrated that the *B. dracunculifolia* leaves extract has relevant *in vivo* hepatoprotective properties, supporting its traditional use. However, more detailed *in vivo* studies are required to establish the safety and clinical relevance of *B. dracunculifolia* leaves extract.
